# The role of carbon capture, utilization, and storage for economic pathways that limit global warming to below 1.5°C

**DOI:** 10.1016/j.isci.2022.104237

**Published:** 2022-04-12

**Authors:** Jenny G. Vitillo, Matthew D. Eisaman, Edda S.P. Aradóttir, Fabrizio Passarini, Tao Wang, Stafford W. Sheehan

**Affiliations:** 1 Department of Science and High Technology and INSTM, University of Insubria, Via Valleggio 9, I-22100 Como, Italy; 2 Department of Electrical & Computer Engineering, Stony Brook University, Stony Brook, NY 11794, USA; 3 Carbfix, Baejarhals 1, 110 Reykjavik, Iceland; 4 Department of Industrial Chemistry “Toso Montanari”, University of Bologna, viale del Risorgimento 4, I-40136 Bologna, Italy; 5 State Key Laboratory of Clean Energy Utilization, Zhejiang University, NO.38, Zheda Road, Hangzhou, 310027 Zhejiang Province, China; 6 Air Company, 407 Johnson Avenue, Brooklyn, NY 11206, USA

**Keywords:** Energy resources, Energy policy, Energy sustainability, Energy Resources

## Abstract

The 2021 Intergovernmental Panel on Climate Change (IPCC) report, for the first time, stated that CO_2_ removal will be necessary to meet our climate goals. However, there is a cost to accomplish CO_2_ removal or mitigation that varies by source. Accordingly, a sensible strategy to prevent climate change begins by mitigating emission sources requiring the least energy and capital investment per ton of CO_2_, such as new emitters and long-term stationary sources. The production of CO_2_-derived products should also start by favoring processes that bring to market high-value products with sufficient margin to tolerate a higher cost of goods.

## Graphical abstract

**Figure:**
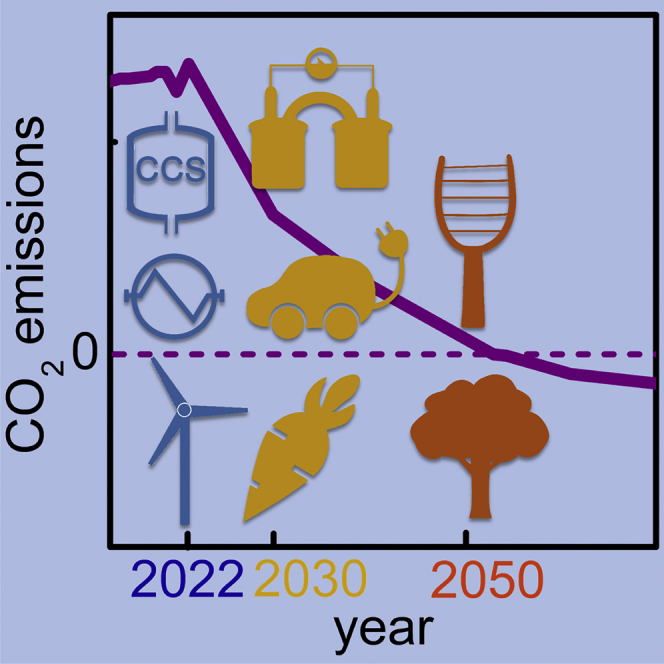

## Introduction

Avoiding the severe impacts of climate change will require a robust framework of policies, certifications, and economic incentives to enable a gradual low-carbon transition in the energy and consumer industries (Otto et al., 2020; Seneviratne et al., 2018; United Nations Framework Convention on Climate Change (UNFCCC), 2015). The Intergovernmental Panel on Climate Change (IPCC) has released a report where the evidence of human influence on climate change is quantified with respect to the increase in severe climate events and the modification of climatic impact-drivers (Intergovernmental Panel on Climate Change, 2021). The near-linear relationship between cumulative anthropogenic CO_2_ emissions and the global mean temperature rise caused by those emissions has been reaffirmed with a high confidence to be 0.45 ± 0.18°C per 1000 Gt_CO2_ (Intergovernmental Panel on Climate Change, 2021). The current state of greenhouse gas emissions suggests that achieving net-zero CO_2_ emissions by mitigation must be accompanied by further CO_2_ removal on a timetable for decarbonization within the coming two to three decades to limit climate extremes (Intergovernmental Panel on Climate Change, 2021).

Climate models, which are mathematical models that are able to describe the physics, chemistry, and biology of processes in the Earth’s atmosphere, land, oceans, and their interaction (Carbon Brief, 2018) are critically important tools to guide policy decisions (Carbon Brief, 2018; Seneviratne et al., 2016, 2018). The information derived from these simulations includes, for example, the global average temperature and trends in weather patterns (Marsh et al., 2007). These models need the evolution of natural forces over time as inputs, but the most recently developed models can also include human effects, such as fossil fuel use. This makes it possible to verify the influence of the various human activities or modification of these activities on the climate. Narratives known as shared socioeconomic pathways (SSPs) (O’Neill et al., 2017; Riahi et al., 2017) are used to describe alternative socioeconomic and technological trajectories. These scenarios correspond to different demographics, technology portfolios, and environmental and natural resources. Including such quantities in the models, either directly or indirectly, helps in evaluating possible policy options for mitigation and adaptation (Hausfather and Peters, 2020; Seneviratne et al., 2016). In this way, postulated evolution in land exploitation and the concentration of greenhouse gases plausible for a defined SSP can constitute the inputs to climate calculations.

These timetables for decarbonization and climate models have not, to date, incorporated deployment scenarios for emerging technologies, such as carbon capture, utilization, and storage (CCUS), which we know are important for limiting global warming to less than 1.5°C. Several CCUS technologies are still at an early stage, where their technical performance at scale and economic impact are relatively unknown. In this Perspective, we discuss the climate models and pathways proposed by the IPCC to minimize greenhouse gas concentrations in the atmosphere in the context of emerging technologies. We outline how new CCUS technologies help keep greenhouse gas emissions on the IPCC’s best-case model by showing a comparable pathway employing CCUS with deployment and greenhouse gas removal driven by technoeconomic value. Lastly, we review how research can help CCUS technologies meet these greenhouse gas removal goals in the context of our experiences deploying CCUS technologies.

### IPCC decarbonization pathways

**Figure 1:**
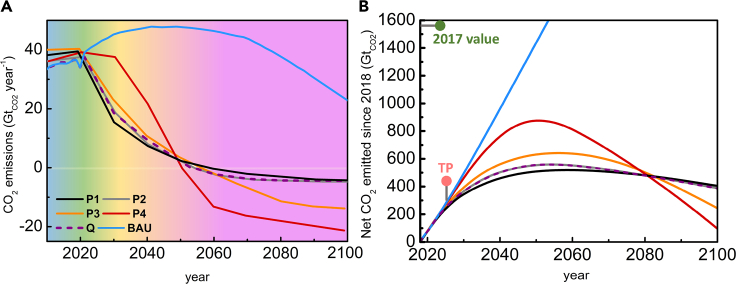
Committed decarbonization pathways (A) CO_2_ emissions per year from 2010 to 2100 according to different scenarios. P1 (black curve), P2 (gray), P3 (orange), and P4 (red) are four possible 1.5°C-committed pathways as reported in Ref (Intergovernmental Panel on Climate Change, 2018). P2, P3, and P4 correspond to three socioeconomic scenarios, whereas P1 is a low energy demand scenario (Intergovernmental Panel on Climate Change, 2018). BAU is a “business-as-usual” scenario, which society is likely to follow given current policies and would correspond to 3°C of warming (Hausfather and Peters, 2020). Q (dashed purple line) is our proposed decarbonization pathway, which accomplishes the P2 emission profile from 2022 and does not require such massive atmospheric scrubbing as in BAU. (B) Cumulative emissions of the pathways shown in part (A) for the 2018–2100 period. The green dot marks the total CO_2_ emissions from 1750 to 2017. The pink dot marks the intersection of P4 with BAU that can be considered as a tipping point (TP) for exceeding 1.5°C in the global temperature increase. The tipping point can be moved forward in time by implementing policies that flatten the 2022–2030 part of the BAU curve.

One of the most policy-relevant results of climate modeling is that the global temperature increase should be kept below 1.5°C when compared to preindustrial values to minimize the cost of adapting to a world with higher atmospheric CO_2_ concentration (Hausfather and Peters, 2020; Intergovernmental Panel on Climate Change, 2018, 2021; Rogelj et al., 2018). Figure 1 shows the global net anthropogenic CO_2_ emission scenarios over the next 80 years as considered in four pathways of the 2018 “IPCC Special Report on 1.5°C” (Intergovernmental Panel on Climate Change, 2018; Rogelj et al., 2018). These pathways are indicated as LED, SSP1, SSP2, and SSP5 in the main report (Rogelj et al., 2018) and P1, P2, P3, and P4, respectively, in the corresponding “Summary for Policymakers” (Intergovernmental Panel on Climate Change, 2018). All of these pathways limit global warming to 1.5°C in 2100. They differ in presenting no (P1 and P2), limited (less than 0.1°C, P3), or higher temperature overshoot (0.1–0.4°C, P4) (Intergovernmental Panel on Climate Change, 2018; Rogelj et al., 2018). These pathways are highly dependent on the projected global energy demand over the next 80 years.

The P1 pathway is obtained assuming low-global energy demand (black curve in Figure 1A), whereas the other three are based on different energetic and socioeconomic scenarios (for more details, see Ref (Rogelj et al., 2018)): (a) a best-case scenario where sustainability is emphasized and fossil fuels are used sparingly (P2, gray curve), (b) a middle-of-the-road scenario with moderate fossil fuel use (P3, orange curve), and (c) a fossil-fuel intensive and high energy demand scenario (P4, red curve). The corresponding CO_2_ emission timeline used in the climate simulations can then be considered as guidelines or thresholds in annual CO_2_ emissions in a committed decarbonization scenario aimed to meet the 1.5°C goal (Heuberger et al., 2018; Intergovernmental Panel on Climate Change, 2018; Rogelj et al., 2018; Sanz-Pérez et al., 2016; Seneviratne et al., 2018; United Nations Framework Convention on Climate Change (UNFCCC), 2015). A possible scenario for “business as usual” CO_2_ emissions (BAU) (Hausfather and Peters, 2020), which is a path society is likely to follow given current policies, is also represented in Figure 1A as a light blue line. This scenario corresponds to the historical emissions up to 2020 (International Energy Agency, 2020; Le Quéré et al., 2021) and to those in the IPCC SSP4-6.0 pathway from 2021 to 2100 (Hausfather and Peters, 2020). The SSP4-6.0 would correspond to a scenario of approximately 3°C of warming (Hausfather and Peters, 2020).

Importantly, to avoid warming beyond 1.5°C, P1-P4 all assume the deployment of carbon dioxide removal from the air (CDR) and in particular of negative emissions technologies (NETs), such as direct air capture (DAC), ocean carbon dioxide removal (ocean CDR), and bioenergy with carbon capture and storage (BECCS) with overall emissions becoming negative by 2050 (Intergovernmental Panel on Climate Change, 2018; Rogelj et al., 2018). NETs are defined as technologies whose operation results in net removal of CO_2_ from the atmosphere rather than just a reduction in CO_2_ emissions. Figure 1B displays the cumulative CO_2_ emissions for each of these scenarios for the years 2018–2100. Each of the P1-P4 curves show a volcano-shaped cumulative-emissions curve with a maxima at around 2050, but the slope of these curves is significantly different on either side of the maxima (see Figure 1B). For years >2050, a more negative slope of the curve indicates a larger number of DAC processes to be placed in operation per year. For P1 and P2, the slope is almost zero: most of the DAC processes will be installed in the 2050–2060 period to reach a constant removal rate of 5 Gt_CO2_ year^−1^. An exemplary breakdown of the IPCC P2 scenario, also referred to as SSP1-19, is shown in Table S1 (Huppmann et al., 2019). These pathways are constructed using the Asia-Pacific Integrated Modeling/Computable General Equilibrium (AIM/CGE) mathematical model that projects the evolution of socioeconomic trends, macroeconomic trends, energy use, and land use to understand net emissions through to 2100.

For P3 and P4, the situation is quite different. For P4, a massive implementation of AC would be required. In fact, in scenario P4 a significant decrease in CO_2_ emissions would start only in 2032. To fight the global temperature increase caused by the addition of 800 Gt_CO2_ into the atmosphere, it would be necessary to reach −20 Gt_CO2_ year^−1^ emissions in 2100, which is a considerable challenge. To give one an idea of the order of magnitude of this value, it coincides with the excess CO_2_ stored in the atmosphere in 2017 (22.4 Gt_CO2_, about 63% of total emissions). The decrease of CO_2_ emissions for 2020 was 2.6 Gt_CO2_ because of the forced lockdown caused by the COVID-19 pandemic (Intergovernmental Panel on Climate Change, 2021; Le Quéré et al., 2021).

Owing to these extreme mitigation and capture requirements, we consider P4 a 1.5°C pathway quite challenging to implement in an economic manner. It would require removing, in the 2050–2100 period, 900 Gt_CO2_ from the atmosphere. Using a back-of-the-envelope calculation, such a process could be possible using a combination of state-of-the-art NET technologies (see Refs (Vitillo, 2015). and (Johnson et al., 2017) for details). Nevertheless, such estimates are obtained using very extreme conditions (e.g., in Ref (Vitillo, 2015), exploitation of the entire Earth’s land area is considered). Because this is clearly an upper bound, we see that the year 2025, the crossing point of BAU and P4 pathways, is a tipping point (TP in Figure 1B) and denotes one of the final practical opportunities to maintain an average global temperature increase below the 1.5°C limit.

In the 2018 IPCC report (Intergovernmental Panel on Climate Change, 2018), P1-P4 scenarios are socioeconomic pathways; that is, the CO_2_ emissions used in the corresponding simulations have been hypothesized considering plausible political, regulatory, cultural, and other socioeconomic changes (for P2, see Table S1 in the Supplemental Data Item). The variables and models that these pathways are derived from can be found in the International Institute for Applied Systems Analysis IAMC 1.5°C Scenario Explorer (Huppmann et al., 2019). Further integrating practical economic and technological constraints to these models is the key to minimizing mitigation cost (van Vuuren et al., 2020) and implementing policies more effectively. Recently, the International Energy Agency (IEA) has released a report aimed at delineating the “Net-zero emissions scenario by 2050” (NZE) (International Energy Agency, 2021). This scenario is aimed at achieving the CO_2_ emissions based on the P2 scenario by the progressive and contemporaneous neutralization of the power, building, transport, industry, and agriculture sectors in the 2021–2050 period.

**Figure 2:**
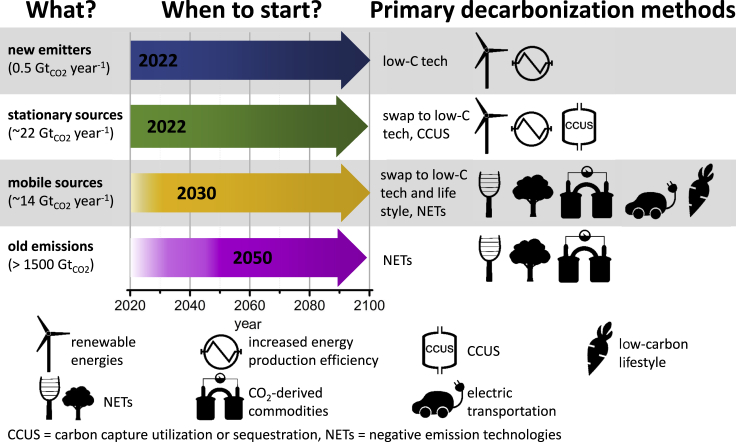
Timetable for the committed decarbonization following the Q pathway The pathway is divided in four phases, ordered in time based on the investment needed per t_CO2_. All the technologies considered in the timetable are commercially deployable now (Bourzac, 2017). It is important to avoid the release of CO_2_ in the atmosphere to maintain the lowest possible cost of the committed decarbonization while deployments of NETs increase and costs are driven down. The time at which each phase of the Q scenario starts should be considered as the last start of each large-scale mitigation action.

If one were to delineate a timetable that uses the IEA scenario but goes further to incorporate CCUS technologies, chances to meet emissions milestones would be further improved. This is achieved by changing the inputs to P2 in a manner that explicitly prioritizes mitigation or removal of emissions based on capital cost and energy requirements. This is shown in Table 1 and the quantitative result of their implementation is shown as scenario “Q”, dashed purple line in Figure 1A with the underlying data shown in Table S2 (for details on the method used to derive the Q scenario please refer to the Supplemental Data Item). Table S3 shows a point-by-point comparison between the P2 and the Q scenarios. The timetable for prioritization per the Q scenario is shown in Figure 2 along with the technologies assumed in each phase. As shown in the Table S2, this scenario uses recent data outlining goals for stationary emitters and air capture within the next 30 years, optimizing for cost (see Tables S4 and S8 in the Supplemental Data Item and Refs (Joos et al., 2016; Joppa et al., 2021; MacDonald et al., 2016)). The colors in the background of Figure 1 further correlate to those used in Figure 2 to differentiate the four phases of the pathway (new emitters, stationary emitters, mobile emitters, and old or legacy emissions).

**Table 1:** Quantitative change in emission goals and description of changes in assumptions for the P2 IPCC pathway, to show the effect of CCUS technology deployment (Q)

Year	P2 Emission Target (Gt_CO2_)	Q Emission Target (Gt_CO2_)	Changes in Assumptions
2021	37.6	37.6	No new stationary sources, begin neutralizing existing stationary sources by mitigation combined with CCUS to make products that displace fossil fuels.
2030	19.0	18.6	CCUS systems deployed at 80% of unmitigable stationary sources and global DAC rollout commences in earnest.
2040	8.2	9.5	Additional dependence on DAC will be required due to unmitigable mobile sources (e.g., airlines), which results in higher emissions in the interim.
2053	0	0	Equilibrium between CO_2_ emitted to the atmosphere and captured by DAC, reforestation, and other methods.
2061	−2.2	−2.2	DAC deployments enable removal of legacy emissions to restore the atmosphere to pre-industrial levels.

Only technologies applicable in the short term (Bourzac, 2017) are considered in the Q pathway. Their order of implementation has been determined by prioritizing technologies that require the least investment per ton of CO_2_ based on today’s estimates (Johnson, 2019; McQueen et al., 2021; Vitillo, 2015). Although such an order for technology deployment may seem obvious, it has not been discussed in literature. Note that the figure presents an average global timetable based on the present emissions. Its aim is to suggest a cadenced pace for the policies on the global level. The time at which each phase of the Q scenario starts should be considered as a maximum time limit for the mitigation of the corresponding emitter categories.

### Future, present, and past CO_2_ emissions

**Figure 3:**
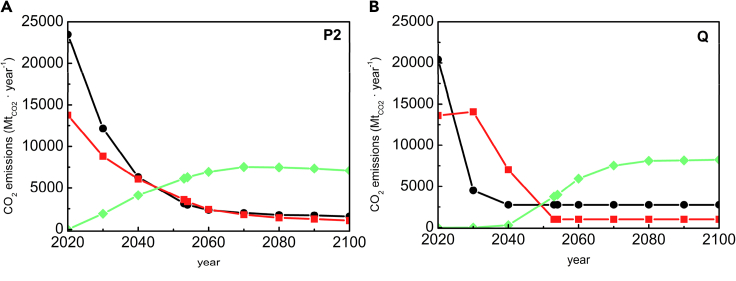
Comparison between P2 and Q emissions pathways (A and B) Emissions from stationary emitters (black circles), mobile emitters (red squares), and negative emissions from air capture technologies (green diamonds) are shown for (A) the P2 pathway, and (B) the Q pathway. Data and calculations are shown in Table S3.

This decarbonization pathway is divided in four phases, wherein most of the emissions reduction comes from the following: (1) phasing out new emitters, (2) mitigation of stationary sources, (3) mitigation of mobile sources, and (4) NETs (see Table S2 in the Supplemental Data Item). The starting time for each phase of Q has been set to follow, at most, the CO_2_ emissions of the P2 pathway, which limits the global temperature increase to 1.5°C and allows for gradually increasing deployment of NETs rather than the requirement of immediate massive deployment. NETs are currently expensive but following gradual implementation pathways such as Q would give scientists, engineers, and entrepreneurs time to reduce costs and scale production, similar to how the cost of solar photovoltaics were driven down in the 50 years between the 1970s and 2020 (Kavlak et al., 2018). Specifically, from 2022 to 2050, the Q pathway uses the P2 emissions profile as a ceiling, but as shown in Figure 2 and Table S3, the Q pathway accomplishes this profile with a different technology portfolio than that assumed by P2 (Rogelj et al., 2018). Figure 3 shows the difference in emission profiles for the P2 and Q pathways, and Table S3 compares them outlining the technological and policy elements that differ between the two. The associated footnotes further highlight how stationary and mobile emitter mitigation can be accelerated in the 2022–2050 time frame with further CCUS technology development. From 2050 to 2100, Q assumes an emission pathway exactly equal to that of P2 and with the same assumed technology portfolio as P2, which includes substantial carbon capture and storage.

We have divided anthropogenic CO_2_ emissions into three main groups: future, present, and past emissions. Each of these three groups has a different mitigation or removal cost. Future emissions are those requiring the lowest cost for their mitigation, amounting to the replacement of emitters with a new technology having a lower (even a negative) carbon footprint (United Nations Framework Convention on Climate Change (UNFCCC), 2021). Although the investment can be significant, it is often a better long-term economic strategy to pay the lower cost of avoiding an emission than to pay the higher cost of capturing it from the atmosphere. New emitters are responsible for a 0.5 Gt_CO2_ increment in emissions each year. Their elimination by the beginning of this year would allow flattening the BAU curve in Figure 1. The first phase of the Q pathway starts by the beginning of 2022: the construction of new emitters ends with an estimated cost of their replacement between 0.3 and 10 $ $t_{CO2}^{-1}$ (United Nations Framework Convention on Climate Change (UNFCCC), 2021).

Existing emitters can be divided into two main categories: stationary sources and mobile sources, accounting for about 60 and 40% of the total emissions (Intergovernmental Panel on Climate Change, 2005), respectively (22 and 14 Gt_CO2_ in 2017). Their mitigation should be completed in 30 years (see green and yellow arrows in Figure 2). Stationary sources, for the most part, include power, steel, petrochemical, and cement plants, whereas mobile sources are primarily transportation. Mitigation of stationary sources in the Q pathway is planned to be 80% (MacDonald et al., 2016) in the 2021–2030 period and 100% in 2050. Stationary sources possess three characteristics that make their mitigation relatively easy: the large amount of CO_2_ emitted per source (major energy industry emitters such as coal and natural gas power plants belong to this category) (Rauner et al., 2020), their relatively low number, and the high CO_2_ concentration at the source (>2 vol %) (Intergovernmental Panel on Climate Change, 2005). As mentioned previously, the most sustainable choice in the long term is the replacement or reconversion of these emitters. For those sources that cannot be replaced or converted, the high CO_2_ concentration in their flue gas makes it energetically favorable to couple them efficiently with CCUS. This agrees with the 2021 IPCC report (Intergovernmental Panel on Climate Change, 2021), and it represents one important difference with respect to the IEA NZE scenario, where the last unabated coal and oil plants are phased out only in 2040 (International Energy Agency, 2021).

Several possible CCUS processes have been already reported that are tailored to a specific stationary source (Gunnarsson et al., 2018; Vatopoulos and Tzimas, 2012; Vitillo et al., 2017). The theoretical minimum energy required for CCUS in a post-combustion system would be only 3.5% of the total produced energy (D'Alessandro et al., 2010), but it can be made lower by increasing the CO_2_ concentration at the inlet and by decreasing the required CO_2_ purity at the output (Joos et al., 2016). However, the energy cost of capturing CO_2_ using standard monoethanolamine (MEA) technology is around 0.37 MWh $t_{CO2}^{-1}$ (Rochelle, 2009), and even with a pathway to 0.20 MWh $t_{CO2}^{-1}$, further research and development toward CO_2_ capture from flue gas is needed to facilitate mitigating stationary emitters at the theoretical minimum. The fact that the 2020 COVID-19 lockdown resulted only in ∼6% reduction in global emissions (Le Quéré et al., 2021), in spite of emissions from transport having gone down considerably, emphasizes the importance of prioritizing mitigation actions from stationary sources such as the energy, construction, and chemical industries (Intergovernmental Panel on Climate Change, 2021).

The mitigation of mobile sources using point-source CCUS processes is not realistic in most cases, because of their large number and small absolute emission quantity per source. For these emitters, replacement, or CDR (nature-based solutions, land management, DAC, BECCS, and ocean CDR) are the only options. In the Q pathway, half of the mobile sources are mitigated in the 2030–2040 period by developments including light vehicle electrification. For the systems that are particularly challenging to be replaced with existing technology (e.g., for planes, responsible for 0.6 Gt _CO2_ emitted each year (Ritchie, 2020) or emissions from the food chain (Berners-Lee, 2010; Eshel et al., 2014; Fuchs et al., 2020)), their mitigation should be done by CO_2_ extraction from the air, together with the remaining 20% of emissions from stationary sources. DAC is relatively energetically demanding compared to point-source capture because of the low volumetric concentration of CO_2_ (418 ppm or 0.0418 vol %) (Keeling, 2022; Tans, 2022). The thermodynamic minimum energy requirement for air capture varies significantly based on inlet and outlet CO_2_ concentration, temperature, and pressure. In several scenarios, the ratio of free energy demand between flue gas capture and direct air capture is not large and varies between 1.06 and 2.93 (Lackner, 2013).

Although this relatively small factor suggests that DAC may be cost effective in the future, DAC costs are still higher than this ratio suggests, in part because it is a newer technology (International Energy Agency, 2021). It has been suggested that its large cost is because of technological challenges more than economy of scale (Keith et al., 2018). As such, the kinetics of CO_2_ capture can be improved, and the large amount of energy required to capture CO_2_ from the air can be decreased by further technology improvement and opportunistic deployment, which would decrease DAC operational expenditures (OpEx). Furthermore, assembly-line manufacturing and large-scale production of individual components would decrease DAC capital expenditures (CapEx). In the Q pathway, reliance on DAC, ocean CDR, and BECCS technologies as CO_2_ mitigation strategies starts in 2050 to provide enough time for research and development as well as large-scale production to decrease its operational cost and capital cost, respectively (Azarabadi and Lackner, 2019; de Lannoy et al., 2018; Eisaman, 2020; Eisaman et al., 2012; Eisaman et al., 2018; National Academies of Sciences, Engineering, and Medicine, 2021). It is important to stress that the deployment of DAC, ocean-CDR, and BECCS technologies should not be delayed. However, it will not be widespread until CapEx and OpEx are improved to the point where renewable energy can power the process in an economically viable manner. Before that, DAC systems deployment remains opportunistic to deploy in locations with abundant renewable electricity and an existing means of utilizing or sequestering CO_2_. One good example is the recently deployed 4000 ton per year scale Climeworks DAC system, driven by geothermal power in Iceland and sequestering CO_2_ in stone using Carbfix systems (Hook, 2021).

Past emissions (purple arrow in Figure 2) are those that remain in the atmosphere, biosphere, and oceans since the start of the Industrial Revolution. They amount to 920 ± 70 Gt _CO2_, increasing by more than 22 Gt_CO2_ each year (Global Carbon Project, 2019). In the BAU scenario, this amount will be doubled in about 30 years (see Figure 1B). Once released in the atmosphere, the emissions can be mitigated only through the application of NETs. The cost of the Q timetable can be roughly estimated by combining the data in Figures 1 and 2 with the costs from Refs (Johnson, 2019; Vitillo, 2015) to be approximately $ 10^13^-10^14^, for 2022–2050 (see Table S7 in the Supplemental Data Item for details). These values are in the range of those estimated by van Vuuren et al. for a 1.5°C-committed pathway (van Vuuren et al., 2020). To put this into a context, the annual gross domestic product of Europe, China, and the United States is of the order of about $ 10^13^ per year each (The World Bank, 2020). The cost of World War II for the United States amounted to $ 4.1 10^12^ in 2021 dollars spread over 5 years (Daggett, 2010). Some studies suggest that the economic cost of climate change adaptation will be lower (Global Commission on Adaptation, 2019) but do not adequately account for long-term effects of climate change beyond 2050. Fundamentally, adaptation deals only with the symptom of the illness, whereas mitigation cures it. An investment in mitigation would thus reduce the future costs in adaptation (Chambwera et al., 2014; Seneviratne et al., 2016, 2018). Adaptation burdens future generations with the social cost of carbon dioxide (SCC), an estimate of the long-term damage done by a ton of CO_2_ emissions in a given year, which would add up to catastrophic levels. The United States Environmental Protection Agency has estimated SCC to be 45 $ $t_{CO2}^{-1}$ with an upcoming revised value expected to be even higher (Wagner et al., 2021). Other estimates allow the SCC value to vary by country and estimate it to be between 10 and 1000 $ $t_{CO2}^{-1}$ (Ricke et al., 2018). The European Community has started the European Green Deal, a $ 10^12^ financial plan over 10 years aimed to foster the investments to make Europe the first climate neutral continent by 2050 (European Commission, 2020). Europe has also recently announced the ban for new fossil fuel cars by 2035. Based on the average age of EU vehicles (10 years) (European Automobile Manufacturers’ Association, 2021), this ban should begin phasing in by 2030, in order to be effective.

**Figure 4:**
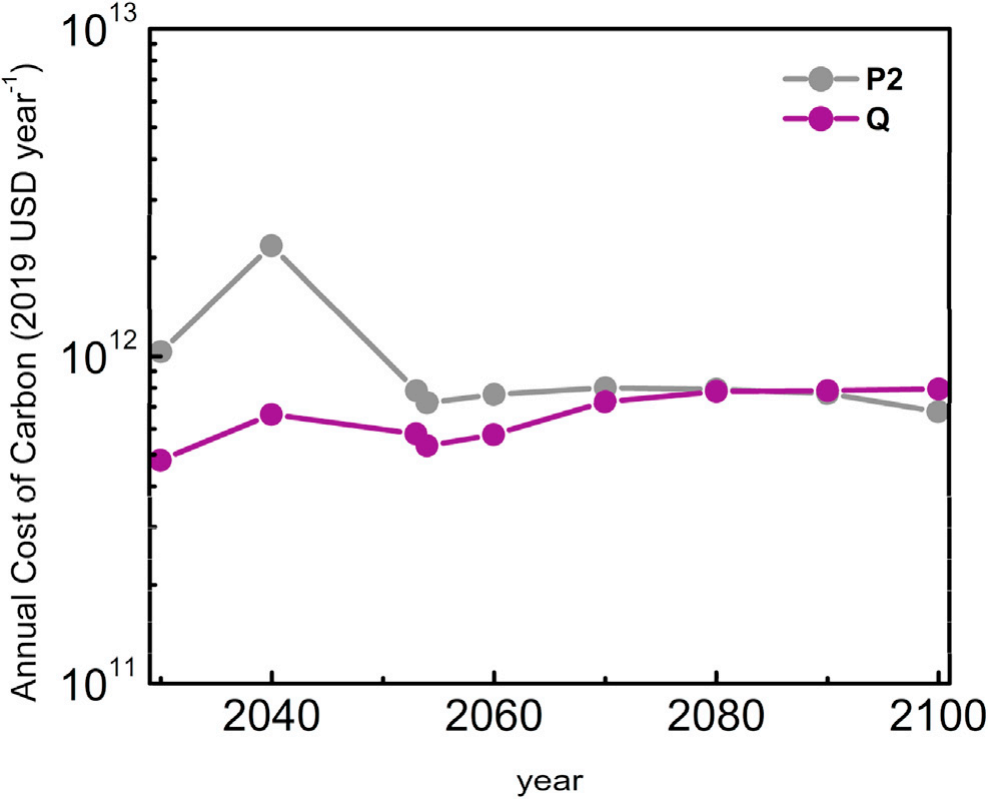
Cost of carbon removal between the different emissions pathways The total annual cost of carbon in 2019 United States Dollars is shown for both the P2 (gray) and Q (purple) pathways. Data and calculations are shown in Table S7. Years <2050: carbon cost as in Table S4. Years ≥2050: carbon cost as in Table S8.

From Figure 1, it is evident that well-timed and swift action is necessary to avoid large costs associated with air capture. C. F. Heuberger et al. (2018) recently quantified this observation using original models, showing that delaying action too long would make useless even the appearance of a “unicorn technology.” Market-driven incentives are critical driving forces to help increase the pace of the transition but are not sufficient by themselves if they do not take into account the cost of carbon removal (Heuberger et al., 2018; Otto et al., 2020). In both P2 and Q pathways, the cost of carbon serves as an indicator of economic incentive that must be put in place to feasibly result in CO_2_ removal. Figure 4 and Table S7 compare the cost of carbon in both pathways and suggest that the Q pathway driven by upfront investment in technology results in a 34.8% reduction in the overall cost of carbon. This result makes intuitive sense; by prioritizing CCUS development today, the technology will be more economically competitive in the future, which lessens the requirements of future regulation. Ultimately, election of governments that enact swift action, as well as adoption of policies and consumer behaviors to limit the increase in average global temperature will require a culture that prioritizes climate action. Beyond government, increased awareness from consumers on the carbon intensity of everyday products would enable collective action that propagates low-carbon products and commodities throughout value chains.

### Critical role of CCUS research

Sequestration will remain a pivotal process all along the mitigation pathway considering that the global demand for CO_2_-derived commodities will likely cover, at its largest development, no more than few Gt_CO2_ per year (Cuéllar-Franca and Azapagic, 2015; D'Alessandro et al., 2010; Mikkelsen et al., 2010; Peplow, 2022; Vitillo, 2015). Nevertheless, CO_2_-derived commodities are often cited as a way to have an additional economic return from the capture process, in addition to offsetting the CO_2_ emissions of production of the equivalent fossil-derived commodity (Hepburn et al., 2019; Peplow, 2022). Conceptually, the easiest way to lower carbon utilization (CU) costs will be to eliminate the capture step (CC or AC), because it is a large fraction of the operational cost of CU processes (Sutter et al., 2019). New efficient materials able to capture and convert CO_2_ selectively from largely diluted mixtures (ideally from air) would represent a breakthrough (Hanusch et al., 2019; Kothandaraman et al., 2016; Sen et al., 2020), as it is the selection of more efficient artificial and synthetic photosynthetic systems (Miller et al., 2020). Lowering the cost of the capture step should be addressed by both process (Sutter et al., 2019) and materials design (Boyd et al., 2019; Danaci et al., 2020; Kim et al., 2020).

For CCS purposes, among the possible processes, research should concentrate on those with low enough capital costs to be coupled with intermittent renewable sources and those that chemically transform CO_2_ into a compound with fixed carbon, as they are more suitable for the permanent storage (Esrafilzadeh et al., 2019; Matter et al., 2016; Snæbjörnsdóttir et al., 2020). Notably, some CCS operations are already able to meet both criteria, transforming captured CO_2_ permanently into minerals underground at a cost lower than the current price of CO_2_ emission allowance in Europe (Esrafilzadeh et al., 2019; Snæbjörnsdóttir et al., 2020). For CU, the initial targets should be CO_2_-derived products with a large profit margin (Chen et al., 2018) instead of those having the larger market, because this can catalyze the fast diffusion of CCUS technologies. Among these products, carbon nanotubes (CNTs) synthetized using electricity coming from solar cells (Johnson et al., 2017; Licht et al., 2016; Ren and Licht, 2016) can represent an economic stepping stone because of their large potential profit margin (100 000-400 000 $ t^−1^). However, they are not yet a widely used industrial feedstock as a large portion of their current market is R&D. Alcohols such as methanol (Marlin et al., 2018) and ethanol (Pace and Sheehan, 2021), are currently being made from CO_2_ on commercially relevant scales and being used in specialty chemicals and consumer goods, respectively (Sarp et al., 2021).

Additional opportunities can also be found in food grade CO_2_ that has a very high cost (ranging from 1515 $ t_CO2_^−1^ for the net CO_2_ gas to 4600 $ t_CO2_^−1^ for the CO_2_ in the cylinders), exceeding the price for CO_2_ capture from air (McQueen et al., 2021) for a total market of 1.4 10^12^ $ year^−1^. Food-grade CO_2_ (99.5% purity) has a market of 0.3 Gt_CO2_ per year. Its dependence on other industrial processes has caused shortages of food-grade CO_2_ in the past and then of carbonized beverages, in periods of high demand (CNBC, 2018). Air capture processes are starting to be used for its production because of the large economic returns and risk mitigation in food markets (Climeworks, 2017; NASA, 2016). Over the longer term, CU products with larger market sizes but smaller profit margins can be targeted to accommodate, at least partly, the large amount of CO_2_ that must be sequestered.

## Conclusions

It cannot be stated too emphatically that the tipping point outlined in Figure 1 is around three years away. It is critical that we continue to invest in research and development for CO_2_ capture, storage, and utilization to drive down the cost of NETs while emphasizing large-scale mitigation strategies in the near-term that enable rapid, economic reduction in CO_2_ emissions. There is historical precedent for global infrastructural change that begins with the most accessible and economic methods, gradually improving to the most sustainable. One of the very industries that CO_2_ removal seeks to mitigate—electricity generation—began in the US by largely mining and burning coal, because it was cheapest and most accessible. As technology, material handling, awareness of pollution, globalization, and other factors occurred, the US electrical grid has gradually moved away from coal toward natural gas. As we move toward the future, the cost of wind and solar are becoming closer to that of natural gas, spurring on a new transition to the most sustainable alternative.

The present pathway suggests a similar trajectory for CO_2_ removal, beginning with the most economic and accessible methods: mitigation and concentrated point-source storage and utilization. These are the technologies that can be used today to address the urgency of climate change. Dilute CO_2_ capture, and specifically DAC, ocean-CDR, and BECCS, is ultimately needed as well. However, it must begin with opportunistic construction of pilot and test plants, as it will take more time for its economics to match that of mitigation and capture from concentrated sources. As the cost per watt of solar photovoltaics was comparatively high in the 1970s, the cost per ton of CO_2_ from DAC, ocean-CDR, and BECCS is high today. This cost differential calls for prioritization of more marketable alternatives in the near-term, as was the case for energy generation when the cost of solar photovoltaics was high. With further development to reduce CO_2_ capture OpEx and sustainable technology production methodologies to lower CapEx improved, policymakers must advocate for NETs for the future as is done for solar photovoltaics today to achieve widespread implementation and meet our climate and energy goals.

## Limitations of the study

The Q pathway was developed following two key principles: (1) it is energetically and economically cheaper to avoid, rather than neutralize, CO_2_ emissions; (2) it is economically favorable to first implement technologies with the lowest cost per ton of CO_2_ mitigated or removed. The ranking of technologies with respect to their cost (and then the order they are implemented in time in Q) obtained from (2) using the data available today can be subjective because of the difficulty of predicting future scientific and technological advancements.

Additional assumptions are reported in the Supplemental Data Item. In particular: (i) for the 2022–2030 period, only 40% of the stationary sources are replaced with carbon-free technologies, whereas the remaining 60% is retrofitted by CCS; (ii) starting from 2040, the stationary sources retrofitted with CCS are substituted with carbon-free technologies at the pace of 5% per year; (iii) the cost associated with DAC is assumed to reach a 5-fold decrease in 2050 with respect to 2020 value according to the estimate in (Sabatino et al., 2021); (iv) the equivalent of 20% of CO_2_ emissions in 2022 associated with the electric grid cannot be neutralized by methods other than carbon dioxide removal from air; (v) DAC rollout starts in earnest in 2030 and shows a 30-fold increase in deployment by 2040 (with respect to 2030) and a 10-fold increase in 2053 (with respect to 2040). For the determination of the total cost of the P2 pathway, we have also assumed that the positive CO_2_ emissions associated with agriculture, forestry, and other land use (AFOLU) are reduced by 50% every ten years while the remainder is neutralized through carbon dioxide removal from air.

Most of these values (highlighted in yellow in the Supplemental Data Item) can be modified by the readers to easily explore scenarios alternative to Q and decrease the bias of the results deriving from our assumptions.

We focused on economic implications and did not consider in our analysis political and social dynamics that can favor the timely adoption of the 1.5°C- committed pathway proposed, because it is outside of our expertise. For these aspects we suggest the interested readers to refer to other studies (see for example Refs (Erans et al., 2022; Heuberger et al., 2018; Otto et al., 2020)).

## Supplemental information

Data S1. Details on the procedure used for the derivation of the Q pathway, related to Figures 1, 2, 3, and 4 The corresponding data for the IPCC P2 pathway, used in the comparison with the Q pathway in the main text, are also reported. Spreadsheet file (∗.XLSX).

## References

[bib1] Azarabadi H., Lackner K.S. (2019). A sorbent-focused techno-economic analysis of direct air capture. Appl. Energy.

[bib2] Berners-Lee M. (2010).

[bib3] Bourzac K. (2017). We have the technology. Nature.

[bib4] Boyd P.G., Chidambaram A., García-Díez E., Ireland C.P., Daff T.D., Bounds R., Gładysiak A., Schouwink P., Moosavi S.M., Maroto-Valer M.M. (2019). Data-driven design of metal–organic frameworks for wet flue gas CO_2_ capture. Nature.

[bib5] Carbon Brief (2018). Q&A: How Do Climate Models Work?. https://www.carbonbrief.org/qa-how-do-climate-models-work.

[bib6] Chambwera M., Heal G., Dubeux C., Hallegatte S., Leclerc L., Markandya A., McCarl B.A., Mechler R., Neumann J.E. (2014). Climate Change 2014: Impacts, Adaptation, and Vulnerability. Part A: Global and Sectoral Aspects. Contribution of Working Group II to the Fifth Assessment Report of the Intergovernmental Panel on Climate Change.

[bib7] Chen C., Kotyk J.F.K., Sheehan S.W. (2018). Progress toward commercial application of electrochemical carbon dioxide reduction. Chem.

[bib8] Climeworks (2017). Climeworks Makes History with World-First Commercial CO_2_ Capture Plant. https://climeworks.com/news/today-climeworks-is-unveiling-its-proudest-achievement.

[bib9] CNBC (2018). Europe Rationing Beer Following Carbon Dioxide Crisis. https://www.cnbc.com/video/2018/06/27/europe-rations-beer-carbon-dioxide.html.

[bib10] Cuéllar-Franca R.M., Azapagic A. (2015). Carbon capture, storage and utilisation technologies: a critical analysis and comparison of their life cycle environmental impacts. J. CO2 Util..

[bib11] D'Alessandro D.M., Smit B., Long J.R. (2010). Carbon dioxide capture: prospects for new materials. Angew. Chem. Int. Ed..

[bib12] Daggett S. (2010). https://fas.org/sgp/crs/natsec/RS22926.pdf.

[bib13] Danaci D., Bui M., Mac Dowell N., Petit C. (2020). Exploring the limits of adsorption-based CO_2_ capture using MOFs with PVSA from molecular design to process economics. Mol. Syst. Des. Eng..

[bib14] de Lannoy C.-F., Eisaman M.D., Jose A., Karnitz S.D., DeVaul R.W., Hannun K., Rivest J.L.B. (2018). Indirect ocean capture of atmospheric CO_2_: Part I. Prototype of a negative emissions technology. Int. J. Greenh. Gas Con..

[bib15] Eisaman M.D. (2020). Negative emissions technologies: the tradeoffs of air-capture economics. Joule.

[bib16] Eisaman M.D., Parajuly K., Tuganov A., Eldershaw C., Chang N., Littau K.A. (2012). CO_2_ extraction from seawater using bipolar membrane electrodialysis. En. Environ. Sci..

[bib17] Eisaman M.D., Rivest J.L.B., Karnitz S.D., de Lannoy C.-F., Jose A., DeVaul R.W., Hannun K. (2018). Indirect ocean capture of atmospheric CO_2_: Part II. Understanding the cost of negative emissions. Int. J. Greenh. Gas Con..

[bib18] Erans M., Sanz-Pérez E.S., Hanak D.P., Clulow Z., Reiner D.M., Mutch G.A. (2022). Direct air capture: process technology, techno-economic and socio-political challenges. Energy Environ. Sci..

[bib19] Eshel G., Shepon A., Makov T., Milo R. (2014). Land, irrigation water, greenhouse gas, and reactive nitrogen burdens of meat, eggs, and dairy production in the United States. Proc. Natl. Acad. Sci. U S A.

[bib20] Esrafilzadeh D., Zavabeti A., Jalili R., Atkin P., Choi J., Carey B.J., Brkljača R., O’Mullane A.P., Dickey M.D., Officer D.L. (2019). Room temperature CO_2_ reduction to solid carbon species on liquid metals featuring atomically thin ceria interfaces. Nat. Commun..

[bib21] European Automobile Manufacturers’ Association (2021). Average Age of the EU Vehicle Fleet, by country. https://www.acea.auto/figure/average-age-of-eu-vehicle-fleet-by-country/.

[bib22] European Commission (2020). A European Green Deal. Striving to Be the First Climate-Neutral Continent. https://ec.europa.eu/info/strategy/priorities-2019-2024/european-green-deal_en.

[bib23] Fuchs R., Brown C., Rounsevell M. (2020). Europe's Green Deal offshores environmental damage to other nations. Nature.

[bib24] Global Carbon Project (2019). CO_2_ Emissions | Global Carbon Atlas. http://www.globalcarbonatlas.org/en/CO2-emissions.

[bib25] Global Commission on Adaptation (2019). Adapt Now: A Global Call for Leadership on Climate Resilience. https://gca.org/wp-content/uploads/2019/09/GlobalCommission_Report_FINAL.pdf.

[bib26] Gunnarsson I., Aradóttir E.S., Oelkers E.H., Clark D.E., Arnarson M.þ., Sigfússon B., Snæbjörnsdóttir S.Ó., Matter J.M., Stute M., Júlíusson B.M., Gíslason S.R. (2018). The rapid and cost-effective capture and subsurface mineral storage of carbon and sulfur at the CarbFix2 site. Int. J. Greenh. Gas Con..

[bib27] Hanusch J.M., Kerschgens I.P., Huber F., Neuburger M., Gademann K. (2019). Pyrrolizidines for direct air capture and CO_2_ conversion. Chem. Commun..

[bib28] Hausfather Z., Peters G.P. (2020). Emissions – the ‘business as usual’ story is misleading. Nature.

[bib29] Hepburn C., Adlen E., Beddington J., Carter E.A., Fuss S., Mac Dowell N., Minx J.C., Smith P., Williams C.K. (2019). The technological and economic prospects for CO_2_ utilization and removal. Nature.

[bib30] Heuberger C.F., Staffell I., Shah N., Mac Dowell N. (2018). Impact of myopic decision-making and disruptive events in power systems planning. Nat. Energy.

[bib31] Hook L. (2021). World’s Biggest ‘direct Air Capture’ Plant Starts Pulling in CO2. https://www.ft.com/content/8a942e30-0428-4567-8a6c-dc704ba3460a.

[bib32] Huppmann D., Kriegler E., Krey V., Riahi K., Rogelj J., Calvin K., Humpenoeder F., Popp A., Rose S.K., Weyant J. (2019). IAMC 1.5°C Scenario Explorer and Data Hosted by IIASA. Integrated Assessment Modeling Consortium & International Institute for Applied Systems Analysis.

[bib33] Intergovernmental Panel on Climate Change (2005).

[bib34] Intergovernmental Panel on Climate Change (2018). Summary for Policymakers of IPCC Special Report on Global Warming of 1.5°C Approved by Governments. https://www.ipcc.ch/2018/10/08/summary-for-policymakers-of-ipcc-special-report-on-global-warming-of-1-5c-approved-by-governments/.

[bib35] Intergovernmental Panel on Climate Change (2021). Climate Change 2021: The Physical Science Basis. Contribution of Working Group I to the Sixth Assessment Report of the Intergovernmental Panel on Climate Change. https://www.ipcc.ch/report/ar6/wg1/.

[bib36] International Energy Agency (2020). Global Energy Review. https://www.iea.org/reports/global-energy-review-2020/global-energy-and-co2-emissions-in-2020.

[bib37] International Energy Agency (2021). Net Zero by 2050. https://www.iea.org/reports/net-zero-by-2050.

[bib38] Johnson J. (2019).

[bib39] Johnson M., Ren J., Lefler M., Licht G., Vicini J., Liu X., Licht S. (2017). Carbon nanotube wools made directly from CO_2_ by molten electrolysis: value driven pathways to carbon dioxide greenhouse gas mitigation. Mater. Today Energy.

[bib40] Joos L., Huck J.M., Van Speybroeck V., Smit B. (2016). Cutting the cost of carbon capture: a case for carbon capture and utilization. Faraday Discuss..

[bib41] Joppa L., Luers A., Willmott E., Friedmann S.J., Hamburg S.P., Broze R. (2021). Microsoft's million-tonne CO_2_-removal purchase - lessons for net zero. Nature.

[bib42] Kavlak G., McNerney J., Trancik J.E. (2018). Evaluating the causes of cost reduction in photovoltaic modules. Energy Policy.

[bib43] Keeling R. (2022). Scripps Institution of Oceanography. http://scrippsco2.ucsd.edu/.

[bib44] Keith D.W., Holmes G., St. Angelo D., Heidel K. (2018). A process for capturing CO_2_ from the atmosphere. Joule.

[bib45] Kim E.J., Siegelman R.L., Jiang H.Z.H., Forse A.C., Lee J.-H., Martell J.D., Milner P.J., Falkowski J.M., Neaton J.B., Reimer J.A. (2020). Cooperative carbon capture and steam regeneration with tetraamine-appended metal–organic frameworks. Science.

[bib46] Kothandaraman J., Goeppert A., Czaun M., Olah G.A., Prakash G.K.S. (2016). Conversion of CO_2_ from air into methanol using a polyamine and a homogeneous ruthenium catalyst. J. Am. Chem. Soc..

[bib47] Lackner K.S. (2013). The thermodynamics of direct air capture of carbon dioxide. Energy.

[bib48] Le Quéré C., Peters G.P., Friedlingstein P., Andrew R.M., Canadell J.G., Davis S.J., Jackson R.B., Jones M.W. (2021). Fossil CO_2_ emissions in the post-COVID-19 era. Nat. Clim. Change.

[bib49] Licht S., Douglas A., Ren J., Carter R., Lefler M., Pint C.L. (2016). Carbon nanotubes produced from ambient carbon dioxide for environmentally sustainable lithium-ion and sodium-ion battery anodes. ACS Cent. Sci..

[bib50] MacDonald A.E., Clack C.T.M., Alexander A., Dunbar A., Wilczak J., Xie Y. (2016). Future cost-competitive electricity systems and their impact on US CO_2_ emissions. Nat. Clim. Change.

[bib51] Marlin D.S., Sarron E., Sigurbjörnsson Ó. (2018). Process advantages of direct CO_2_ to methanol synthesis. Front. Chem..

[bib52] Marsh P.T., Brooks H.E., Karoly D.J. (2007). Assessment of the severe weather environment in North America simulated by a global climate model. Atmos. Sci. Lett..

[bib53] Matter J.M., Stute M., Snæbjörnsdottir S.Ó., Oelkers E.H., Gislason S.R., Aradottir E.S., Sigfusson B., Gunnarsson I., Sigurdardottir H., Gunnlaugsson E. (2016). Rapid carbon mineralization for permanent disposal of anthropogenic carbon dioxide emissions. Science.

[bib54] McQueen N., Gomes K.V., McCormick C., Blumanthal K., Pisciotta M., Wilcox J. (2021). A review of direct air capture (DAC): scaling up commercial technologies and innovating for the future. Prog. Energy.

[bib55] Mikkelsen M., Jørgensen M., Krebs F.C. (2010). The teraton challenge. A review of fixation and transformation of carbon dioxide. Energy Environ. Sci..

[bib56] Miller T.E., Beneyton T., Schwander T., Diehl C., Girault M., McLean R., Chotel T., Claus P., Cortina N.S., Baret J.-C., Erb T.J. (2020). Light-powered CO_2_ fixation in a chloroplast mimic with natural and synthetic parts. Science.

[bib57] NASA (2016). CO_2_ Recovery System Saves Brewers Money, Puts Bubbles into Beer. https://spinoff.nasa.gov/Spinoff2016/cg_3.html.

[bib58] National Academies of Sciences, Engineering, and Medicine (2021).

[bib59] O’Neill B.C., Kriegler E., Ebi K.L., Kemp-Benedict E., Riahi K., Rothman D.S., van Ruijven B.J., van Vuuren D.P., Birkmann J., Kok K. (2017). The roads ahead: narratives for shared socioeconomic pathways describing world futures in the 21^st^ century. Glob. Environ. Change.

[bib60] Otto I.M., Donges J.F., Cremades R., Bhowmik A., Hewitt R.J., Lucht W., Rockström J., Allerberger F., McCaffrey M., Doe S.S.P. (2020). Social tipping dynamics for stabilizing Earth’s climate by 2050. PNAS.

[bib61] Pace G., Sheehan S.W. (2021). Scaling CO_2_ capture with downstream flow CO_2_ conversion to ethanol. Front. Clim..

[bib62] Peplow M. (2022). The race to upcycle CO_2_ into fuels, concrete and more. Nature.

[bib63] Rauner S., Bauer N., Dirnaichner A., Dingenen R.V., Mutel C., Luderer G. (2020). Coal-exit health and environmental damage reductions outweigh economic impacts. Nat. Clim. Change.

[bib64] Ren J., Licht S. (2016). Tracking airborne CO_2_ mitigation and low cost transformation into valuable carbon nanotubes. Sci. Rep..

[bib65] Riahi K., van Vuuren D.P., Kriegler E., Edmonds J., O’Neill B.C., Fujimori S., Bauer N., Calvin K., Dellink R., Fricko O. (2017). The Shared Socioeconomic Pathways and their energy, land use, and greenhouse gas emissions implications: an overview. Glob. Environ. Change.

[bib66] Ricke K., Drouet L., Caldeira K., Tavoni M. (2018). Country-level social cost of carbon. Nat. Clim. Change.

[bib67] Ritchie H. (2020). Climate Change and Flying: What Share of Global CO2 Emissions Come from Aviation?. https://ourworldindata.org/co2-emissions-from-aviation.

[bib68] Rochelle G.T. (2009). Amine scrubbing for CO_2_ capture. Science.

[bib69] Rogelj J., Shindell D., Jiang K., Fifita S., Forster P., Ginzburg V., Handa C., Kheshgi H., Kobayashi S., Kriegler E., Mundaca L., Séférian R., Vilariño M.V., Masson-Delmotte V., Zhai P., Pörtner H.-O., Roberts D., Skea J., Shukla P.R., Pirani A., Moufouma-Okia W., Péan C., Pidcock R., Connors S., Matthews J.B.R., Chen Y., Zhou X., Gomis M.I., Lonnoy E., Maycock T., Tignor M., Waterfield T. (2018). Global Warming of 1.5°C. An IPCC Special Report on the impacts of global warming of 1.5°C above pre-industrial levels and related global greenhouse gas emission pathways, in the context of strengthening the global response to the threat of climate change, sustainable development, and efforts to eradicate poverty.

[bib70] Sabatino F., Grimm A., Gallucci F., van Sint Annaland M., Kramer G.J., Gazzani M. (2021). A comparative energy and costs assessment and optimization for direct air capture technologies. Joule.

[bib71] Sanz-Pérez E.S., Murdock C.R., Didas S.A., Jones C.W. (2016). Direct capture of CO_2_ from ambient air. Chem. Rev..

[bib72] Sarp S., Hernandez S.G., Chen C., Sheehan S.W. (2021). Alcohol production from carbon dioxide: methanol as a fuel and chemical feedstock. Joule.

[bib73] Sen R., Goeppert A., Kar S., Prakash G.K.S. (2020). Hydroxide based integrated CO_2_ capture from air and conversion to methanol. J. Am. Chem. Soc..

[bib74] Seneviratne S.I., Donat M.G., Pitman A.J., Knutti R., Wilby R.L. (2016). Allowable CO_2_ emissions based on regional and impact-related climate targets. Nature.

[bib75] Seneviratne S.I., Rogelj J., Séférian R., Wartenburger R., Allen M.R., Cain M., Millar R.J., Ebi K.L., Ellis N., Hoegh-Guldberg O. (2018). The many possible climates from the Paris Agreement’s aim of 1.5°C warming. Nature.

[bib76] Snæbjörnsdóttir S.Ó., Sigfússon B., Marieni C., Goldberg D., Gislason S.R., Oelkers E.H. (2020). Carbon dioxide storage through mineral carbonation. Nat. Rev. Earth Environ..

[bib77] Sutter D., van der Spek M., Mazzotti M. (2019). 110th anniversary: evaluation of CO_2_-based and CO_2_-free synthetic fuel systems using a net-zero-CO_2_-emission framework. Ind. Eng. Chem. Res..

[bib78] Tans P. (2022). NOAA/ESRL. http://www.esrl.noaa.gov/gmd/ccgg/trends/.

[bib79] The World Bank (2020). World Development Indicators. http://datatopics.worldbank.org/world-development-indicators/.

[bib80] United Nations Framework Convention on Climate Change (UNFCCC) (2015). Adoption of the Paris Agreement FCCC/CP/2015/L.9/Rev.1. http://unfccc.int/resource/docs/2015/cop21/eng/l09r01.pdf.

[bib81] United Nations Framework Convention on Climate Change (UNFCCC) (2021). Carbon Offset Platform. https://offset.climateneutralnow.org/allprojects.

[bib82] van Vuuren D.P., van der Wijst K.-I., Marsman S., van den Berg M., Hof A.F., Jones C.D. (2020). The costs of achieving climate targets and the sources of uncertainty. Nat. Clim. Change.

[bib83] Vatopoulos K., Tzimas E. (2012). Assessment of CO_2_ capture technologies in cement manufacturing process. J. Clean. Prod..

[bib84] Vitillo J.G. (2015). Magnesium-based systems for carbon dioxide capture, storage and recycling: from leaves to synthetic nanostructured materials. RSC Adv..

[bib85] Vitillo J.G., Smit B., Gagliardi L. (2017). Introduction: carbon capture and separation. Chem. Rev..

[bib86] Wagner G., Anthoff D., Cropper M., Dietz S., Gillingham K.T., Groom B., Kelleher J.P., Moore F.C., Stock J.H. (2021). Eight priorities for calculating the social cost of carbon. Nature.

